# Metabolic rate and critical thermal maximum *CT_max_* estimates for westslope cutthroat trout, *Oncorhynchus clarkii lewisi*

**DOI:** 10.1093/conphys/coac071

**Published:** 2022-12-21

**Authors:** Eva C Enders, Travis C Durhack

**Affiliations:** Institute National de la Recherche Scientifique, Centre Eau Terre Environnement, Québec Québec, G1K 9A9, Canada; Fisheries and Oceans Canada, Freshwater Institute, Winnipeg Manitoba, R3T 2N6, Canada

**Keywords:** westslope cutthroat trout, respirometry, metabolic rate, critical thermal maximum, Aerobic scope

## Abstract

Global warming is changing the thermal habitat of cold-water freshwater fishes, which can lead to decreased fitness and survival and cause shifts in species distributions. The Alberta population of westslope cutthroat trout (*Oncorhynchus clarkii lewisi*) is listed as ‘Threatened’ under the Canadian *Species at Risk Act*. The major threats to the species are the alteration in habitat and water flow, competition and hybridization with non-native trout species and climate change. Here, we conducted (i) intermittent-flow respirometry experiments with adult native westslope cutthroat trout and non-native rainbow trout (*Oncorhynchus mykiss*) and (ii) critical thermal maximum experiments (*CT_max_*) with adult westslope cutthroat trout to obtain valuable input data for species distribution models. For both species, standard metabolic rate (SMR) was lower at 10°C compared to 15°C and westslope cutthroat trout had higher SMR than rainbow trout. Although there were inter-specific differences in SMR, forced aerobic scope (using a standardized chase protocol) was different at 10°C, but no significant differences were observed at 15°C because of relative smaller differences in maximum metabolic rate between the species. *CT_max_* of westslope cutthroat trout acclimated to 10°C was 27.0 ± 0.8°C and agitation temperature was 25.2 ± 1.0°C. The results from this study will inform and parametrize cumulative effects assessments and bioenergetics habitat modelling for the recovery planning of the species.

## Teaser

We measured metabolic rates of adult native westslope cutthroat trout and non-native rainbow trout and the critical thermal maximum of adult westslope cutthroat trout. The results will inform and parametrize models used in the recovery planning of westslope cutthroat trout.

## Introduction

Regarding warming temperatures and climate change, the biogeographical principle of ‘adapting, migrating or going extinct’ is highly relevant for cold-water fishes (Munday *et al.*, 2017). Populations can adapt to changing temperature regimes through evolution or phenotypic plasticity and/or by migrating to more suitable thermal habitat. The effects of temperature on the metabolic rates, aerobic scope and upper critical temperature limits of cold-water fishes are of particular interest and therefore widely studied (Imholt *et al.*, 2011; Durhack, 2020; Morrison *et al.*, 2020; Macnaughton *et al.*, 2021) as they represent valuable input parameters for various modelling approaches including, e.g. bioenergetics habitat models (Naman *et al.*, 2020), species distribution models (Pandit *et al.*, 2017), minimum viable population analyses (van der Lee and Koops, 2020) and cumulative effects assessments (DFO, 2019a). In particular, aerobic scope, which is defined as the scope of aerobic metabolic rates above the maintenance levels scope (Fry, 1947) and estimated as the difference between the maximum metabolic rate and the standard metabolic rate (SMR), provides insight into a fish’s upper capacity to supply oxygen for life processes such as reproduction, growth and activity in addition to its minimum level necessary to maintain homeostasis (Nelson, 2016). The aerobic scope generally follows a curve, peaking at an optimal temperature (*T_opt_*) before decreasing with increasing water temperature (Fry, 1947) where eurythermal species that are thermal generalists have a high aerobic scope over a broad range of temperatures whereas stenothermal species that are thermal specialists have an aerobic scope optimum over a narrow range of temperatures (Durhack *et al.*, 2021; Macnaughton *et al.*, 2021). Quantifying how a species physiologically reacts at different temperatures helps us understand its thermal thresholds and assists with delineating important thermal critical habitats.

Upper thermal limits in fish can be estimated using non-lethal, acute temperature experiments (e.g. Critical Thermal Maximum (*CT_max_*) and agitation temperature (*T_ag_*)). *CT_max_* is a useful, repeatable estimate of the sublethal upper temperature limit of a species and defined as the temperature when an individual looses equilibrium and is unable to maintain an upright position in the experimental chamber (Becker and Genoway, 1979). *CT_max_* is likely several degrees higher than the temperature a species can tolerate over prolonged time periods. Therefore, the temperature where a fish is showing an avoidance response and is trying to escape from increasing water temperature (McDonnell and Chapman, 2015) is thought to be more ecologically relevant. The temperature, which triggers an avoidance behaviour at which the fish abandon their habitat to seek shelter in colder habitat, e.g. tributaries or thermal refugia, is defined as the agitation temperature (*T_ag_*) (Firth *et al.*, 2021; McDonnell and Chapman, 2015). The results of *CT_max_* experiments are also crucial as input variables for various modelling efforts used to inform management decisions, such as bioenergetics habitat modelling, species distribution modelling and cumulative threat risk assessments.

Historically, westslope cutthroat trout (*Oncorhynchus clarkii lewisi*) occurred in mountain lakes, headwater streams and into large rivers in southern Alberta, eastern Washington, Idaho, western Montana, eastern Oregon and northwestern Wyoming but currently the subspecies displays a fragmented distribution over large portions of its historic range (Behnke, 1992; Shepard *et al.*, 2005). Several populations of westslope cutthroat trout are considered at risk under the Canadian *Species at Risk Act* and the US *Endangered Species Act*. The survival and recovery of threatened westslope cutthroat trout are jeopardized by the cumulative effects of increasing water temperature due to climate change, competition and hybridization from non-native trout species (i.e. rainbow trout), recreational fishing and habitat destruction due to development. Subsequently, this study is very timely for filling some of the knowledge gaps in the thermal limits of westslope cutthroat trout and may be important for provincial and federal entities managing the species by providing information on the physiology and habitat limitations of this species. The upper lethal temperature for westslope cutthroat trout has been described as lower (19.7°C) than for rainbow trout (24.4°C), but both species had similar optimum growth temperatures (13.6 and 13.1°C, respectively; Bear *et al.*, 2007). A wide range of *CT_max_* values have been reported in the literature depending on cutthroat trout subspecies, population, life stage and acclimation (De Staso and Rahel, 1994; Wagner *et al.*, 2001; Underwood *et al.*, 2012). Subsequently, we analyzed the metabolic rates and aerobic scope at two ecologically relevant temperatures (10°C and 15°C) of adult westslope cutthroat trout (*Oncorhynchus clarkii lewisi*), a species at risk in Canada, and adult rainbow trout (*Oncorhynchus mykiss*) an introduced congeneric species that competes with westslope cutthroat trout for resources. We also assessed upper critical temperature limits of adult westslope cutthroat trout via *T_ag_*, *CT_max_* and the ‘*CT_max_*–*T_ag_* window’, the difference between *CT_max_* and *T_ag_* (Wells *et al.*, 2016). A large *CT_max_*-agitation window points toward an individual ceasing regular behavior and seeking refuge from the temperature increases earlier than an individual with a smaller window and provides an indication of the thermal buffer of the fish to escape thermally stressful conditions before losing equilibrium (Wells *et al.*, 2016; Firth *et al.*, 2021).

The specific objectives of this study were to (i) evaluate the effect of temperature on the metabolic rates (i.e. standard and maximum metabolic rates, aerobic scope) of westslope cutthroat trout and rainbow trout using intermittent-flow respirometry and (ii) analyze thermal thresholds for westslope cutthroat trout using behavioral *CT_max_* experiments.

## Materials and methods

We achieved our objectives by estimating the SMR, spontaneous maximum metabolic rate (MMR_s_) and forced maximum metabolic rate (MMR_f_) of westslope cutthroat trout and rainbow trout under two temperature acclimations and calculating the corresponding spontaneous aerobic scope (AS_s_) and forced aerobic scope (AS_f_). We also estimated *CT_max_* and agitation temperature on westslope cutthroat trout acclimated to 10°C.

## Experimental Animals

Westslope cutthroat trout eggs and milt were collected from the Fording River system in British Columbia, Canada (N 50°13′42.96″, W 114°51′39.71″) and fertilized at the aquatic fish holding facility of the Fisheries and Oceans Canada’s Freshwater Institute (Winnipeg, Manitoba, Canada) in July 2012. Fish used for respirometry experiments were first-generation (F1) gametes collected from the Fording River System. Fish used for *CT_max_* experiments were the second generation (F2) bred from the Fording River system gametes. Juvenile rainbow trout were obtained from Lyndon Fish Hatcheries (New Dundee, Ontario, Canada) in April 2014. Upon arrival at Fisheries and Oceans Canada’s Freshwater Institute, fish weighed ~1 g.

All fish were held in 600-l flow-through tanks using de-chlorinated city water maintained at water temperatures of 10°C and 15°C and exposed to a 12:12 diurnal lighting regime with gradual light changes at 07:00 and 19:00 to mimic dawn and dusk. Fish were fed 0.5% of body weight using Hi-Pro Trout food #3 pellet (Hi-Pro Feeds, Okotoks, Alberta, Canada). Holding and experimental procedures were approved by the Freshwater Institute Animal Care Committee (Animal Use Protocols: FWI-ACC-AUP 2014-001, FWI-ACC-AUP 2015-001, FWI-ACC-AUP-2020-07) following the guidelines and recommendations outlined by the Canadian Council on Animal Care.

## Intermittent-flow respirometry

### System setup

An intermittent-flow respirometry system with four respirometry chambers (cylindrical plexiglass chambers; 14 cm in diameter × 45 cm in length, 7070 ml volume; Loligo^®^ Systems Tjele, Denmark) was used to estimate SMR and MMR_f_ of westslope cutthroat trout (AutoResp™ 2.2.0; Loligo^®^). The chambers were submerged in temperature-controlled tanks (300 l; 10.0°C ± 0.1°C or 15.0°C ± 0.1°C, depending on treatment) using water baths (Lauda Alpha, models RA 24 and A 24, Lauda-Königshofen, Germany). Oxygen concentrations in the tank were kept above 90% oxygen saturation using air stones.

The respirometry chambers were connected to two pumps (Universal 1048 Eheim, Deizisau, Germany) by non-toxic, Polyvinyl Chloride tubing. One pump recirculated water throughout the system and across an adjacent oxygen probe vessel. The other pump brought oxygenated water from the tank to the chamber and restored <90% oxygen saturation during the open flush phase. The pumps were controlled by AutoResp software (version 2.2; Loligo Systems, Tjele, Denmark) to produce a suite of opened and closed water circulation phases in the chambers. During the closed phase, only the recirculating pump was activated and no water or oxygen exchange with water outside of the respirometer chamber occurred. The oxygen depletion caused by fish and bacterial respiration was measured during this closed phase. The four oxygen probes (optical sensor dipping sensor, DP-PSt3-L2.5-ST10-YOP; precision ±0.05 mg O_2_∙l^−1^, PreSens, Regensburg Germany) were relayed to a multi-channel oxygen meter (OXY-4 mini, PreSens, Regensburg Germany) and run by the AutoResp software recording the oxygen concentrations.

### Experimental procedure

Intermittent-flow respirometry experiments were conducted between January and March 2016. A total of 39 westslope cutthroat trout (mean ± S.D. body mass = 423.2 ± 104.7 g) and 32 rainbow trout (mean ± S.D. body mass = 438.9 ± 96.1 g) were used in the respirometry experiments. Fish were fasted for at least 24 h before experiments and were randomly selected from the treatment tank and transferred to a respirometry chamber. Fish were exposed to the same 12:12 diurnal cycle as during the fish-holding and isolated from any external stimuli with a dark curtain draped around all edges of the tank. Activity in the room where experiments were conducted was kept to a minimum.

Estimates of SMR, MMR_f_, MMR_s_, AS_f_ and AS_s_ were obtained for each fish. Estimation of SMR and MMR_s_ was conducted first over a 48 h period (flush period, 5 min; wait period, 1 min; measuring period, 2–5 min). The measurement period varied depending on temperature and fish body mass to ensure that oxygen conditions remained normoxic in the chambers and to avoid oxygen concentrations below 7.6 mg O_2_·l^−1^, a critical value that may affect respiration rates (Tang *et al.*, 2000). Consequently, a whole respirometry cycle lasted for 8–11 min. Following SMR, fish were exhausted using a standardized chase protocol of 5 min manual chase protocol consisting of a 1 min chase in a circular bucket, 3 min turning the fish over and 1 min holding the fish out of the water (similar to Roche *et al.*, 2013). At the end of this 5 min chase, fish were not capable of burst swimming, a sign of exhaustion (Milligan, 1996). Immediately after chasing, fish were returned to the respirometry chamber and three oxygen consumption rate measurements were taken (*ṀO_2_*; see data analysis below). MMR_f_ was defined as the single highest *ṀO_2_* of the three estimates observed immediately after the chase procedure. MMR_s_ was calculated as the highest single *ṀO_2_* observed during the 24 h experiment (excluding MMR_f_ measurements). Aerobic scopes between both SMR and MMR_f_ (AS_f_) and SMR and MMR_s_ (AS_s_) were calculated by subtracting the SMR from the MMR_f_ and MMR_s_, respectively. Background bacterial oxygen demand (BOD) was estimated in each chamber by taking 10 min measurements of empty respirometry chambers immediately before and after each trial. The value of these BOD measurements was then subtracted from SMR and MMR estimates to adjust for BOD.

**Figure 1:**
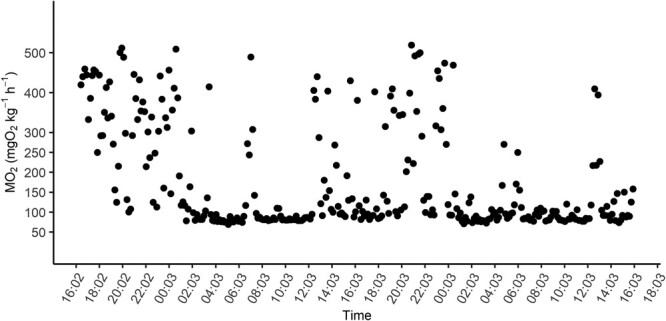
Oxygen consumption rate (*ṀO_2_*) of representative adult Westslope Cutthroat Trout over a 48 h respirometry experiment.

### Critical thermal maximum *CT*_*max*_

#### System setup

*CT_max_* experiments were conducted from 30 November to 4 December 2020. For the *CT_max_* experiment, two 200-l tanks were held at 10°C with aeration and two pumps (Universal 1048, Eheim, Deizisau, Germany) for circulation to ensure uniform heating. Each tank contained four 300 W titanium heating elements (TH-0300S titanium heaters, Finnex, Chicago, USA), which were used to heat the water during trials. Preliminary testing was conducted to select a water depth (24 cm) that ensured heaters consistently heated the water at a rate of 0.3°C·min^−1^. Westslope cutthroat trout were fasted for 24 h and placed in the same respirometry chambers as used in intermittent-flow respirometry experiments with the end caps removed and instead covered with soft mesh to allow for better water exchange with the tank water. Two fish per tank were left to acclimate overnight in the covered tank before starting a respective trial.

#### Experimental procedure

A total of 20 westslope cutthroat trout (mean ± S.D. body mass = 503.5 ± 140.9 g) were used for *CT_max_* experiments. A given *CT_max_* trial consisted of removing the tank cover, fish were then given 10 min to recover from being disturbed before the heaters were turned on and the trial began. Water temperatures were increased at a rate of 0.3°C min^−1^ and fish were constantly observed to monitor for abnormal behavior. The trial length was recorded, with temperature and dissolved oxygen levels being verified every 5 min to ensure that heating rates were constant and that dissolved oxygen saturations were >90%. Fish were monitored for two behavioural reactions, i.e. agitation temperature and *CT_max_*. Agitation temperature was defined as the temperature at which fish displayed a sustained (>5 s) escape response behavior (McDonnell and Chapman, 2015). This escape response behavior was defined as ‘curling’ and ‘bursting’ movements being displayed. Curling was defined as the fish’s body being curved in a C shape as a result of attempting to turn around in the chamber and bursting behavior was defined as bursts of energetic swimming and was generally observed as sustained swimming into the netting enclosing the ends of the chamber. Both types of behavior were taken as an attempt to escape increasing water temperatures and subsequently the agitation temperature reflects physiological changes causing stress due to elevated water temperatures (McDonnell and Chapman, 2015).

*CT_max_* was defined as a loss of equilibrium (LOE), which was defined as the endpoint of the trial (Beitinger *et al.*, 2000; Morrison *et al.*, 2020). Once LOE was reached, fish were removed from the treatment tank and quickly transferred to a recovery bath held at 10°C. Fish were left in the recovery bath overnight before being returned to a general population tank.

### Data analysis

The oxygen consumption rate (*ṀO_2_*) was calculated as follows:

(1) \begin{align*} \dot{M}{O}_2=\frac{\Delta {O}_2}{\Delta t}\bullet \frac{\left({V}_R-{V}_F\right)}{M}, \end{align*}

where *ṀO_2_* is the mass-specific oxygen consumption rate (mg O_2_·kg^−1^·h^−1^), $\Delta {O}_2$ is the decline in oxygen content (mg/l) in the chamber, $\Delta t$is the time elapsed during the closed measuring period (h), consequently $\frac{\Delta {O}_2}{\Delta t}$ is the oxygen depletion slope, *V_R_* the water volume in the respirometer system (l), V*_F_* the fish volume (l) and M the fish body mass (kg).

Visually inspecting *ṀO_2_* by time graphs showed that westslope cutthroat trout had an habituation time of 10 ± 5 h. An example of typical patterns of *ṀO_2_* of westslope cutthroat trout during respirometry experiments can be seen in Fig. 1.

SMR was estimated as the *q_0·2_* or mean of the lowest normal distribution of all *ṀO_2_* estimates during the last 38 h of the experiment (Chabot *et al.*, 2016). MMR_s_ was calculated as the highest single *ṀO_2_* observed during the 48 h experiment (excluding MMR_f_ measurements; Svendsen *et al.*, 2014). MMR_f_ was defined as the single highest *ṀO_2_* of the three estimates observed immediately after the chase procedure (Svendsen *et al.*, 2012). Aerobic scopes between both, SMR and MMR_s_ (AS_s_) and SMR and MMR_f_ (AS_f_) were calculated by subtracting the SMR from MMR_s_ and MMR_f_, respectively (Svendsen *et al.*, 2012).

The mean temperatures where sustained agitation (>5 s), LOE and the difference between the two for all fish tested were used as the agitation temperature, *CT_max_* and *CT_max_*-agitation window (Beitinger *et al.*, 2000).

### Statistical analysis

Analyses of metabolic rates and *CT_max_* experiments were conducted using R version 4.0.2 (R Foundation for Statistical Computing, 2020) and R Studio version 1.3.1056 (Rstudio PBC, 2020). Experimental data (SMR, MMR_s_, MMR_f_, AS_s_, AS_f_) was analyzed using two-way ANOVAs, with species and temperature as variables. Student’s *t*-tests were used to compare differences between MMR_f_ and AS_f_, MMR_s_ and AS_s_, as well as between-sex differences for *CT_max_*. Residual plots were examined for normality and homogeneity among treatments and in all cases were found to meet assumptions. The full analysis of metabolic rate estimate comparisons can be found in Supplementary Table S1.

## Results

### Metabolic rates

**Figure 2:**
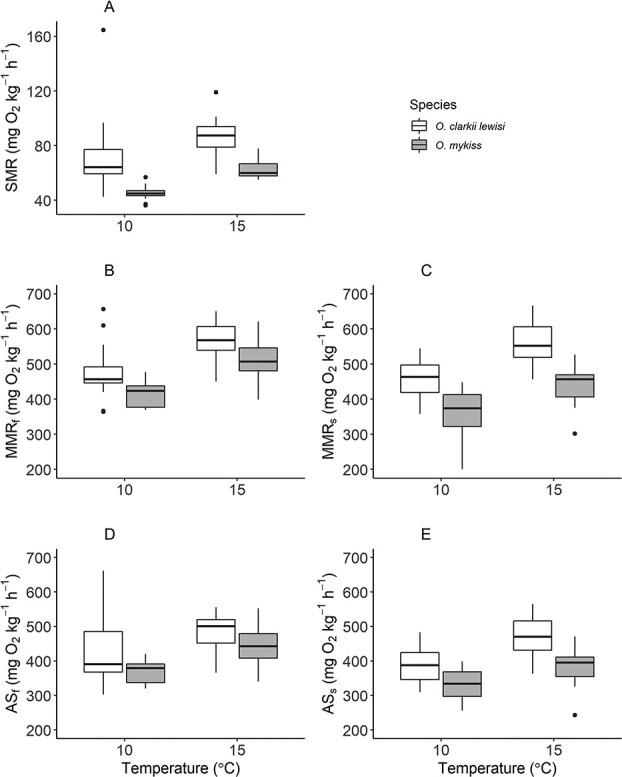
Metabolic rate estimates of adult Westslope Cutthroat Trout (Oncorhynchus clarkii lewisi) and Rainbow Trout (Oncorhynchus mykiss) tested at 10 and 15°C. (A) Standard metabolic rate (SMR), (B) Forced maximal metabolic rate (MMRf), (C) Spontaneous maximal metabolic rate (MMRs), (D) Forced aerobic scope (ASf), (E) Spontaneous aerobic scope (ASs).

Westslope cutthroat trout and rainbow trout had higher metabolic rates at 15°C compared to 10°C (Table 1; Fig. 2). SMR was different both between temperatures (*P* < 0.001) and species (*P* < 0.001). The intra-specific comparison found no difference in forced maximum metabolic rate (MMR_f_) between temperatures for westslope cutthroat trout (*P* = 0.059). However, MMR_f_ was higher at 15°C compared to 10°C for rainbow trout (*P* < 0.001). Inter-specific comparisons showed lower MMR_f_ for rainbow trout than westslope cutthroat trout at both 10°C and 15°C (*P* = 0.002 and *P* = 0.010, respectively).

**Table 1:** Mean and standard deviation estimates of metabolic rates of adult Westslope cutthroat trout and rainbow trout at 10°C and 15°C

	Westslope cutthroat trout		Rainbow trout	
Temperature (°C)	10	15	10	15
SMR (mg O_2_·kg^−1^·h^−1^)	71.83 ±6.00	86.54 ±2.90	45.32 ±1.30	62.18 ±1.73
MMR_s_ (mg O_2_·kg^−1^·h^−1^)	459.04 ± 11.90	560.62 ± 12.99	366.56 ± 16.25	444.85 ± 14.80
MMR_f_ (mg O_2_·kg^−1^·h^−1^)	507.69 ± 24.59	564.95 ± 14.28	414.14 ±8.74	508.70 ± 14.85
AS_s_ (mg O_2_·kg^−1^·h^−1^)	387.21 ± 11.22	474.26 ± 12.71	321.25 ± 15.30	382.68 ± 14.58
AS_f_ (mg O_2_·kg^−1^·h^−1^)	435.86 ± 25.55	478.59 ± 14.86	368.83 ±7.86	441.88 ± 15.51

Similarly, both species had higher MMR_s_ at 15°C compared to 10°C (*P* < 0.001 and *P* = 0.002, respectively) and westslope cutthroat trout had higher MMR_s_ than rainbow trout. MMR_f_ was higher than MMR_s_ for both species, with MMR_f_ being different between test temperatures for rainbow trout (RNTR, 10°C: *P* = 0.003; 15°C: *P* = 0.004). However, these differences were not significantly different for westslope cutthroat trout (WSCT, 10°C: *P* = 0.087; 15°C: 0.764).

Forced aerobic scope (AS_f_) was not different between temperatures for westslope cutthroat trout (ANOVA; *P* = 0.170) but it was higher at 15°C for rainbow trout (*P* < 0.001). AS_f_ was higher for westslope cutthroat trout at 10°C than rainbow trout (*P* = 0.026); however, no difference was observed at 15°C (*P* = 0.098). Spontaneous aerobic scope was found to be different at 10°C and 15°C for both species (WSCT: *P* < 0.001; RNTR: *P* = 0.007), as well as different between species at both test temperatures (10°C: *P* = 0.001; 15°C *P* < 0.001). Similar to MMR, AS_f_ and AS_s_ were not found to be different between 10°C and 15°C for westslope cutthroat trout (*P* = 0.087 and *P* = 0.764, respectively); however, AS_f_ was higher for rainbow trout than AS_s_ at both 10 and 15°C (*P* = 0.003 and *P* = 0.009, respectively).

### Thermal limits

**Figure 3:**
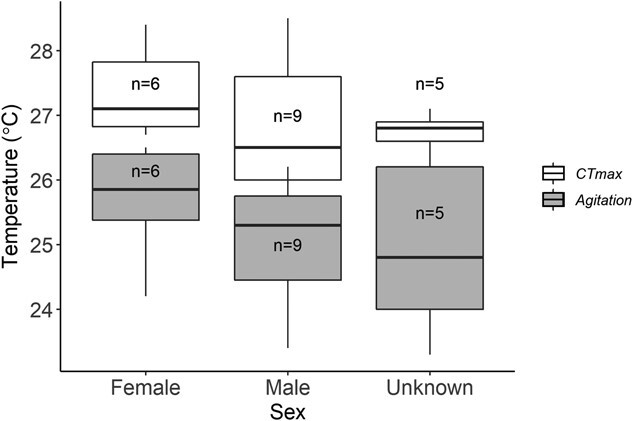
Critical Thermal Maximum (CTmax) and agitation temperature (Tag) estimates of adult Westslope Cutthroat Trout (Oncorhynchus clarkii lewisi) based on sex. Gray boxes display the Tag estimates, white boxplots display *CT_max_* estimates. No difference was found for any metric between sexes (P = 0.200, *P* = 0.300, and *P* = 0.500, respectively)

The critical thermal maximum of adult westslope cutthroat trout was 27.0 ± 0.8°C (mean ± S.D.), while the mean ± S.D. agitation temperature was 25.2 ± 1.0°C. The *CT_max_*-agitation window for westslope cutthroat trout was relatively small with 1.8 ± 0.9°C. Only one fish did not recover from the *CT_max_* experiments, with the mortality occurring shortly after being placed into the recovery bath. Of the 20 fish tested, we were able to identify the sex of 15 fish (9 males, 6 females, 5 unknown). *T_ag_* and *CT_max_* were slightly higher for females than males, while the *CT_max_*- *T_ag_* was slightly lower, with mean ± S.D. agitation temperature, *CT_max_*, and *CT_max_*- *T_ag_* window for females being 25.7 ± 0.8°C, 27.4 ± 0.6°C, and 1.7 ± 0.7°C, respectively, and 25.0 ± 0.9°C, 26.8 ± 1.0°C, and 2.0 ± 0.8°C, respectively, for males (Fig. 3). There was no difference between sexes for any of the comparisons (*P* = 0.200, *P* = 0.300, and *P* = 0.500, respectively).

## Discussion

Due to climate change, westslope cutthroat trout may experience shifts in the distributions of their thermal habitat (Williams *et al.*, 2009; Heinle *et al.*, 2021). In order to simulate changes to species distributions and inform recovery strategies and actions, particularly for species at risk, it is important to understand a species’ physiology and thermal limits. The results from the intermittent-flow respirometry experiments on westslope cutthroat trout and its non-native competitor rainbow trout provide not only a parametrization of the temperature dependency of metabolic rates and aerobic scope but also valuable insights into inter-specific differences. Westslope cutthroat trout have a higher SMR and MMR than rainbow trout. Interestingly, at lower temperatures of 10°C westslope cutthroat trout had also a higher AS than rainbow trout, but no significant differences were observed at 15°C indicating a higher metabolic capacity of westslope cutthroat trout at lower temperatures (Fry, 1947; Pörtner and Farrell, 2008). Subsequently, the higher AS at lower temperature may indicate an advantage of westslope cutthroat trout over rainbow trout that could be compromised with increasing water temperatures due to climate change (Crozier *et al.*, 2021).

A comparison of our mass-specific metabolic rate data to a previous intermittent-flow respirometry study on juvenile westslope cutthroat trout at the same temperatures showed a lower mean for SMR, MMR_f_ and AS_f_ in the adult fish in comparison to juveniles. A decrease in mass-specific metabolic rates is to be expected in larger, older individuals as growth and basic energetic demands decrease as organisms age (Hunt von Herbing and White, 2002; Killen *et al.*, 2010). When comparing the rainbow trout metabolic rates to previous studies (Blake and Chan, 2006; Murray *et al.*, 2017; Ekström *et al.*, 2018), a similar decrease in metabolic rates as the fish increase in size was observed.

Albeit some evidence that chase protocols may underestimate MMR (Hvas and Oppedal, 2019; Raby *et al.* 2020), Little *et al.* (2020) demonstrated that for salmonids the here applied standardized chase protocol provided similar MMR estimates than those obtained with swim tunnels. Interestingly in our study, the MMR obtained using the standardized chase protocol resulted in similar rates to those estimated when analysing at the fish’s voluntarily displayed spontaneous MMR similar to the results by Zhang *et al.* (2020).

In regards to the critical thermal maximum experiments of westslope cutthroat trout acclimated to 10°C, *CT_max_* of westslope cutthroat trout was slightly lower (i.e. 27.0 ± 0.8°C) than for the two co-occurring introduced, non-native salmonid species (i.e. rainbow trout and brook trout (*Salvelinus fontinalis*)) in the native range of westslope cutthroat trout. For example, estimates of *CT_max_* for rainbow trout acclimated to ~10°C range from 27.6°C to 28.5°C (Lee and Rinne, 1980; Carline and Machung, 2001), while estimates for brook trout range from 28.2°C to 28.7°C for fish acclimated to ~10°C (Lee and Rinne, 1980; Carline and Machung, 2001). While both of these introduced species are considered to have a warmer water preference than westslope cutthroat trout are thought to, the similar *CT_max_* values and small *CT_max_*-agitation window exhibited by adult westslope cutthroat trout in our study suggest an ability to handle warmer water temperatures than previously assumed, as has also been suggested by thermal preference testing by Macnaughton *et al.* (2018, 2021). Westslope cutthroat started to become agitated at a temperature of 25.2 ± 1.0°C. While the high agitation temperature suggests an ability to withstand warmer water temperature for a short period before avoidance behaviour sets in, the small *CT_max_*-agitation window (1.8 ± 0.9°C) may indicate detrimental sub-lethal thermal effects are initiating if the fish is unable to move to cooler thermal refugia quickly.

Further understanding of the thermal performance of westslope cutthroat trout will help conservation efforts of the species, both for understanding areas to protect as Critical Habitat, as well as to better understand how future climate change scenarios may affect the species distributions. More specifically, the results from this study will inform and parametrize cumulative effects assessments and minimal viable population analyses for the recovery planning of the species that are laid out in species’ recovery strategy and action plan and help delineate their critical habitat, respectively (DFO, 2019b).

## Funding

This research was funded by Fisheries and Oceans Canada’s Species at Risk Program to ECE.

## Data Availability

Data for this paper can be found at https://doi.org/10.5683/SP3/LFJ5NZ

## Conflict of Interest

Not used.

## Supplementary Material

Web_Material_coac071
