# Supplementary material for: Metabolic rate and critical thermal maximum CTmax estimates for westslope cutthroat trout, Oncorhynchus clarkii lewisi
Source: Conserv Physiol. 2022 Dec 21;10(1):coac071. doi: 10.1093/conphys/coac071 (PMC9773365; doi:10.1093/conphys/coac071)
Supplement: Web_Material_coac071 [file web_material_coac071.zip › supplementary_tablesS1S2_coac071.docx]

**Supplementary Table S1:** Statistical analysis of ANOVA comparisons of metabolic rate estimates for standard metabolic rate (SMR), forced maximum metabolic rate (MMR_f_), spontaneous maximum metabolic rate (MMR_s_), forced aerobic scope (AS_f_), and spontaneous aerobic scope (AS_s_) for Westslope Cutthroat Trout (WSCT) and Rainbow Trout (RNTR). Response variable indicates whether comparisons were performed across species (species) or within species, across temperatures (Temp).

|  | Response | *P* | *F* | d.f. |
| --- | --- | --- | --- | --- |
| 10 °C SMR | Species | <0.001 | 15.854 | 1,33 |
| 15 °C SMR | Species | <0.001 | 43.939 | 1,33 |
| 10 °C MMR_f_ | Species | 0.002 | 11.142 | 1,33 |
| 15 °C MMR_f_ | Species | 0.010 | 7.459 | 1,31 |
| 10 °C MMR_s_ | Species | <0.001 | 21.919 | 1,33 |
| 15 °C MMR_s_ | Species | <0.001 | 37.586 | 1,34 |
| 10 °C AS_f_ | Species | 0.026 | 5.409 | 1,33 |
| 15 °C AS_f_ | Species | 0.098 | 2.913 | 1,30 |
| 10 °C AS_s_ | Species | 0.001 | 12.572 | 1,33 |
| 15 °C AS_s_ | Species | <0.001 | 21.353 | 1,33 |
| WSCT SMR | Temp | 0.030 | 5.034 | 1, 37 |
| WSCT MMR_f_ | Temp | 0.059 | 3.813 | 1, 34 |
| WSCT MMR_s_ | Temp | <0.001 | 32.344 | 1, 37 |
| WSCT AS_f_ | Temp | 0.170 | 1.965 | 1, 34 |
| WSCT AS_s_ | Temp | <0.001 | 24.873 | 1, 37 |
| RNTR SMR | Temp | <0.001 | 61.597 | 1, 29 |
| RNTR MMR_f_ | Temp | <0.001 | 30.127 | 1, 30 |
| RNTR MMR_s_ | Temp | 0.002 | 11.509 | 1, 30 |
| RNTR AS_f_ | Temp | <0.001 | 18.342 | 1, 29 |
| RNTR AS_s_ | Temp | 0.007 | 8.403 | 1, 29 |

**Table S2.** Settings used during the intermittent-flow respirometry experiments to estimate SMR and MMR
